# Puncture of Hæmarthros

**Published:** 1880-04

**Authors:** Richard Volkman, Herman Mynter


### PUNCTURE OF HÆMARTHROS.—BY RICHARD VOLKMAN.

FROM THE GERMAN BY HERMAN MYNTER, M. D.

Dr. Riedel's investigations (*Deutsche Zeitschrift fuer Chirurgie* Bd. xii, S. 447), as to whether the blood coagulates in hæmarthros, when and under what circumstances the coagulation takes place, when and under what circumstances absorption takes place, are of great practical value. The author found in a large number of punctures the blood perfectly or almost perfectly fluid, when in cases of traumatic hydarthros the trocar was introduced during the first three days. Once after six days, 50 ccm., and three times after eight days, respectively 50, 80 and 100 ccm. dark fluid blood were removed without traces of coagulas, while in a large number of other cases of puncture between the fourth and eight day, the principal part of the blood was fluid, but some coagulas were removed through the trocar, and others left behind in the joint. Once in a case of transverse fracture of the patella (5 days old) with broad diastasis of the fragments, and the joint perfectly filled, he found the blood coagulated in such a degree, that not a drop could be aspirated.

Against Riedel's belief, that even large coagulas of blood are quickly absorbed, the author mentions a case, in which he amputated fourteen weeks after the accident, and found the synovial cavity still filled with a large amount of coagulas, which adhered firmly to the synovial membrane, and not a drop of synovial fluid was found in the joint.

The author has advocated the use of the puncture of the knee-joint in cases of traumatic hamarthros, since the introduction of the antiseptic treatment of wounds. He states that anchylosis with total obliteration of the joint may occur in cases in which the coagulated blood is quickly organized. It does not occur often, and only provided a uniform layer of clot is deposited on the inner surface of the synovial membrane and the secretion of synovia is suppressed perfectly by it. Volkman verified this statement once in case of transverse fracture of the patella. Eleven months afterwards, when the patient died of some other disease, the knee-joint was found anchylosed in a straight position, the cartilage intact but united with each other and with the synovial membrane, by a continuous layer of fibrous tissue (1—2 lines thick), so that not the least movement was possible.—*Centralblatt fuer Chirurgie.*

